# Dr. Juan Garcia Puron

**Published:** 1912-08

**Authors:** 


Dr. Juan Garcia Puron died in his birth-place, Llanes, Asturias, Spain, June 9, aged 58. He was known to the American profession by a long career with the Appleton Publishing Co.

